# How do people with knee osteoarthritis use osteoarthritis pain medications and does this change over time? Data from the Osteoarthritis Initiative

**DOI:** 10.1186/ar4286

**Published:** 2013-09-04

**Authors:** Sarah R Kingsbury, Elizabeth MA Hensor, Ceara AE Walsh, Marc C Hochberg, Philip G Conaghan

**Affiliations:** 1 Leeds Institute of Rheumatic and Musculoskeletal Medicine, University of Leeds and NIHR Leeds Musculoskeletal Biomedical Research Unit, Chapel Allerton Hospital, Chapeltown Road, Leeds, LS7 4SA, UK; 2 University of Maryland School of Medicine, 655 West Baltimore Street, Baltimore, MD 21201-1559, USA

**Keywords:** Medications, knee osteoarthritis, Osteoarthritis Initiative

## Abstract

**Introduction:**

The aim of this analysis was to describe comprehensively the cross-sectional and longitudinal patterns of analgesic and nutraceutical medication use for knee osteoarthritis (OA) in a contemporary US cohort and to investigate associated demographic and clinical factors.

**Methods:**

Baseline, 12, 24 and 36 month data were obtained retrospectively from the National Institutes of Health Osteoarthritis Initiative. Participants had symptomatic radiographic knee OA. Multiple binary logistic regression models identified characteristics independently associated with the use of analgesics or nutraceuticals.

**Results:**

We included 987 subjects (55.9% female, mean age 61.5 years, 71.0% white). At baseline, 68.2% reported frequent use of a conventional analgesic or nutraceutical for joint pain (for more than half of the previous month). Non-prescription non-steroidal anti-inflammatory drugs (NSAIDs) were the most frequently reported medications (26.8%), even in those more than 75-years old. Multiple conventional analgesics were used by 11.9%. Frequent analgesic use was more likely in women (odds ratio (OR) 1.8 (95% confidence interval (CI) 1.3 to 2.3)) and people with more pain (moderate 1.7 (1.2 to 2.4); severe 3.1 (2.1 to 4.7)); nutraceutical use was less likely in non-whites (0.4 (0.3 to 0.6)), those more than 74-years old (0.6 (0.3 to 0.9)) and those with comorbidities (0.6 (0.5 to 0.9)) and more likely in people with Kellgren-Lawrence (KL) grade 4 (2.2 (1.5 to 3.3)). Overall there was no change in the proportion of participants frequently using prescription or over the counter (OTC) analgesics at 36 months, although most people had changed medication type; of those using a traditional analgesic at baseline approximately one third were still using the same type at 36 months (ranging from 26.2% of baseline prescription NSAID users to 40.6% of baseline acetaminophen users). All participants reporting baseline analgesic use also reported 36 month analgesic use. Female participants (OR 95% CI 1.2 to 3.2, *P *= 0.009), those with high body mass index (1.2 to 4.8, *P *= 0.010) and those with moderate (1.6 to 2.6, *P *= 0.090) or severe (1.8 to 12.0, *P *= 0.002) baseline pain were more likely to use pain medication during the 36 month follow-up period; participants more than 75-years old were less likely (0.2 to 1.0, *P *= 0.053).

**Conclusions:**

Most people with knee OA used pharmacological therapies frequently, and use appeared to be according to American College of Rheumatology recommendations. Change in medication type used was common. Persistent non-prescription NSAID use in older people is an area of concern.

## Introduction

Osteoarthritis (OA) has a profound impact on overall quality of life [1-4]. In the United States, OA is the most prevalent joint disease and the leading cause of chronic disability [5]; 26.9 million people 25-years-old or over have clinical OA of at least one joint [6], costing an estimated $89.1 billion per year [7]. Poorer outcome in terms of pain and function has been linked to risk factors such as female sex, high body mass index (BMI) and African-American ethnicity [8-11].

Various treatment options have been proven effective in reducing OA pain [12] and current guidelines for the contemporary management of hip or knee OA recommend the use of both non-pharmacological and pharmacological therapies [13-16]. In addition to traditional therapies, there are increasing reports of the use of nutraceuticals (defined as 'foodstuffs which provide health benefits in addition to their basic nutritional value') for the treatment of OA, although current guidelines do not recommend them [14,15]. Despite a marked increase in nutraceutical use for all indications over the past decade [17], few studies have investigated their use by people with OA.

A number of studies have examined how specific classes of therapies are used by people with OA and the relationship of use with various demographic factors, including age, gender and race. These studies suggest, for example, that African-Americans are prescribed fewer analgesics [18-21], opioid use declines in older patients [21] and women use more analgesics and a higher number of medications [22]. However, few recent studies have comprehensively examined the overall pattern of use and frequency with which OA pharmacological therapies are prescribed and factors associated with their use. In particular, there has been little research regarding the use of nutraceuticals or the use of combinations of therapy by individuals. Identification of factors associated with use of pharmacological therapy is the first step toward improving OA therapy for these populations.

Because the prevalence of knee OA increases with age, the efficacy and safety/tolerability of prescribed drugs must be carefully considered; more than 90% of patients with OA are at increased gastrointestinal (GI) and/or cardiovascular (CV) risk. Use of NSAIDs and COX-2 inhibitors (coxibs) has been associated with a range of increased CV and GI complications [23-26] and are not recommended in those more than 75-years-old [15]. A recent study of non-steroidal anti-inflammatory drug (NSAID) prescription suggested that in more than half of OA patients, NSAID prescription was not in accordance with these guidelines [27]. Examining medication use in this age group, where treatment options are more limited, is, therefore, particularly pertinent.

The aim of this study was, therefore, to comprehensively describe analgesic and nutraceutical medication use at baseline and over time in a contemporary US cohort and its relationship with age, sex, race, BMI, co-morbidities, Kellgren-Lawrence (KL) grade and severity of pain.

## Methods

Data used in the preparation of this article were obtained from the Osteoarthritis Initiative (OAI) database, a publicly available multi-centre population-based observational cohort study of knee OA which is available for public access [28]. Specific datasets used are detailed in Additional File 1, Methods. The OAI cohort is composed of three groups, the Progression (*n *= 1,390) and Incidence (*n *= 3,285) subcohorts and the Non-exposed Control group (*n *= 122). The Progression subcohort consists of individuals (age 45 to 79 years) with symptomatic tibiofemoral knee OA in at least one knee at baseline and was the focus of the current study. Symptomatic tibiofemoral knee OA is defined in the OAI as 1) participant report of frequent knee symptoms (defined as aching/pain/stiffness in or around the knee) on most days for ≥1 month during the past 12 months and 2) radiographic evidence of tibiofemoral knee OA defined as the presence of an Osteoarthritis Research Society International (OARSI) atlas osteophyte grade 1 to 3, equivalent to Kellgren and Lawrence (KL grade) ≥2, on fixed flexion radiograph based on the individual clinic readings. Follow-up data are currently available at 12, 24 and 36 months. For this analysis we used baseline and 36 months data, except for the analysis of frequent use of medication for pain, aching or stiffness at any point over 36 months of follow-up, for which the 12 and 24 month data were also used.

Baseline assessment included age, sex and ethnicity. Co-morbid medical conditions were assessed by a self-reported version of the Charlson comorbidity index [29]. Self-reported global knee pain severity during the past 30 days was assessed using a 0 to 10 numerical rating scale (NRS). An inventory of all prescription medication used in the past 30 days was collected at the baseline visit. Subjects were asked to bring in or identify all prescription medication taken in the preceding 30 days; subjects were not asked to bring in over-the-counter (OTC) medications. Use of prescription gastro-protective agents was extracted from this medication inventory. Participants were also asked whether they had used prescription analgesics in the last 30 days, and whether they had used classes of prescription or OTC medications (acetaminophen, NSAIDs, coxibs, opioids) and/or nutraceuticals (glucosamine, chondroitin, methylsulfonylmethane (MSM), doxycycline or S-adenosylmethionine (SAMe)) for more than half of the days of the previous month, specifically for pain, aching or stiffness in their knee (classed as frequent use). The design of the OAI medication questionnaire allowed us to distinguish between prescription and OTC NSAID use and these were considered different 'types' of analgesic, but it was not possible to determine whether or not acetaminophen had been prescribed.

Further details on the specific databases accessed during this study and the OAI exclusion criteria are provided in Additional File 1, Methods. We excluded subjects who had knee replacements because they could have been taking medication for pain due to knee replacement. We also excluded those with missing data due to drop out, incomplete patient-reported arthritis pain medication data or incomplete baseline demographic and/or clinical data.

Statistical analysis was performed using SPSS version 19.0.2. Baseline characteristics evaluated for their association with medication use included sex; race (white, non-white, the latter category comprising African-Americans, Asians and 'non-white: other'); age (grouped into three bands according to risk factors for medication use: under 65 years, 65 to 74 years and over 74 years); severity of pain in the most painful knee (as defined by their highest score on NRS or right side if equal, divided into three groups at the lower and upper quartiles of the distribution 0 to 3, 4 to 7, 8 to 10); KL grade in the most painful knee (≤1, 2, 3 or 4); presence of co-morbidities; and BMI (<26, 26 to 30, >30).

A number of different measures of patient-reported medication use were examined:

1. Any use of prescription analgesics (any; type unspecified) in the month prior to assessment (yes/no).

2. Frequent use (that is, on more than half of the days) in the month prior to assessment (yes/no) of a) traditional analgesics (acetaminophen, prescription NSAIDs, OTC NSAIDs, coxibs, opioids) and/or b) nutraceutical medications (glucosamine, chondroitin, MSM, doxycycline or SAMe).

3. The total number of analgesic types (acetaminophen, prescription NSAID, OTC NSAID, coxibs, opioid) used frequently in the month prior to assessment.

4. The change in the use of analgesics at 36 months; whether participants who were taking at least one analgesic at baseline were still taking the same drug types, whether they had switched or added drug types, or had reduced the number of types they were taking.

5. Frequent use of medication for pain, aching or stiffness at any point over 36 months of follow-up (yes/no), the derivation of which included the intervening annual assessments (12 and 24 months).

Multiple binary logistic regression models were used to identify characteristics that were independently associated at baseline with the odds of having used medication within the past month and with the odds of having used pain medication over 36 months of follow-up. Independent variables were entered simultaneously into the model in one block; in a second block interaction terms age*KL grade; age*comorbidity; sex*pain; race*pain were entered using backwards selection and were retained if *P *<0.05. We focussed on these specific interactions since they have previously been reported to be associated with pain [30,31]. The specific interactions listed were determined *a priori*; backwards selection was used to ensure that only those which were significant were retained in the model, thus maximising the accuracy and interpretability of the results. A generalized linear model assuming a negative binomial distribution and using a log link was also created to identify factors associated with the number of prescription or OTC analgesic types that participants reported using frequently in the month prior to baseline.

Summaries of specific medication types are presented to give an indication of whether similar trends were observed for all analgesics and/or nutraceuticals. These results are considered exploratory due to the small numbers of participants taking some types of medication, and no formal analyses were performed to identify predictors of use of individual medication types.

All statistical tests were two-tailed; family-wise Holm-Bonferroni corrections for multiple comparisons were made, separating baseline modelling of medication use from 36-month changes; for multivariable models only the overall significance of each model was included in the correction. Following correction the threshold for significance testing at the 5% level was set to *P *= 0.05 at baseline and *P *= 0.006 for changes at 36 months.

## Results

**Figure 1:**
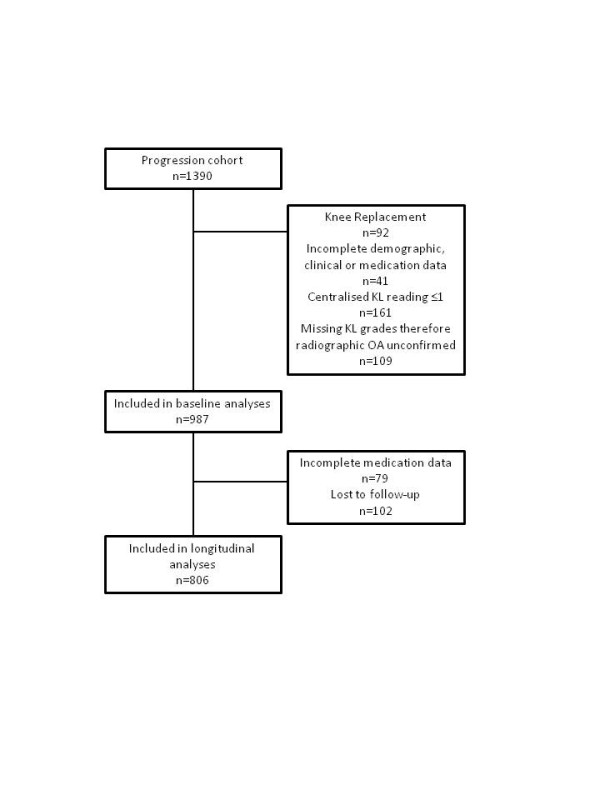
**Flow chart outlining inclusion of participants into the study**.

Of the 1,390 subjects in the Progression cohort, 987 were included in the baseline analysis and 806 in the longitudinal analysis. Reasons for exclusion are presented in Figure 1.The likelihood of data being incomplete was not related to the majority of demographic and clinical variables; a slightly higher proportion of non-white subjects were excluded due to incomplete data at baseline (8.0% versus 2.2%). Non-white subjects were not substantively more likely to have incomplete data at follow-up (8.7% versus 7.3%) but were slightly more likely to be lost to follow-up (14.1% versus 8.1%).

The study population (*n *= 987) were 55.9% female and 71.0% white; 89.5% of the non-white group were African-American. Mean ± SD (range) age was 61.5 ± 9.0 (45 to 79) years and BMI was 30.3 ± 4.9 (18.2 to 48.7) kg/m^2^. The majority of participants met the American College of Rheumatology (ACR) definition for an OA diagnosis which requires articular knee pain for most days of the prior month, radiographic evidence of osteophytes on joint margins and either crepitus on active motion, motion stiffness less than 30 minutes duration or age more than 38 years [32]. All had reported radiographic OA at screening and were aged >38, 864 [87.7%] stated that they had experienced pain on most days during the previous month and a further 8.7% recorded pain NRS scores greater than 2 units in their most painful knee in the previous month. KL grade in the most painful knee was grade 2 in 38.3% of subjects; grade 3 in 44.8% and grade 4 in 16.9%. The median (IQR) number of medications (taken for all indications) was 3.0 (2.0 to 5.0).

That some subjects in the OAI progression cohort had KL grades <2 despite the radiographic inclusion criteria is likely related to disagreement between the initial OAI enrolment centre assessment and subsequent centralised KL readings. The OAI have compared the individual clinic radiographic readings against a blinded, centralised reading of a randomly-selected subset and reported substantial but not perfect agreement (weighted Kappa 0.71 (0.63 to 0.79)).

### Patterns of medication use at baseline

Medication use at baseline is summarised in Table 1. At baseline two-thirds of subjects (673/987, 68.2%) had used either a conventional analgesic (defined as OTC NSAID, prescription NSAID, acetaminophen, prescription coxib or opioid) or nutraceutical medication (glucosamine, chondroitin, MSM, doxycycline or SAMe) for joint pain or arthritis for more than half of the days in the month prior to the visit. Non-prescription (OTC) NSAIDs were the most commonly used analgesic, and the agent used most commonly in isolation (63.0% of OTC NSAID users, 167/265). Few patients were using opioids (3.3% compared with 6.5% of the patients excluded due to total knee replacement). Only a small number of participants had received an intra-articular injection of either steroid or hyaluronan.

**Table 1:** Severity of symptoms and medications used during the month prior to assessment at baseline and 36 months

Variable		Baseline All patients N = 987	Baseline Followed up N = 806	36 months Followed up N = 806
Pain NRS in most painful knee, mean ± SD		5.5 ± 2.4	5.4 ± 2.4	4.8 ± 2.8
Prescription analgesic used at any time		30.1%	29.4%	24.2%^a^
Prescription or OTC analgesic used frequently^b^		47.8%	47.9%	42.9%
Nutraceuticals used frequently^b^		41.3%	43.4%	36.0%^a^
Number of medications Analgesics	0:	52.2%	52.1%	57.1%
used frequently^b^	1:	36.0%	37.6%	33.3%
	2:	9.9%	8.8%	7.8%
	≥3:	1.9%	1.5%	1.8%
Nutraceuticals	0:	58.7%	56.6%	64.0%^a^
	1:	4.1%	4.2%	4.1% ^a ^
	2:	30.1%	31.5%	22.8% ^a^
	≥3:	6.8%	7.7%	9.1% ^a^
Patient-reported medication use:				
Acetaminophen^b^		13.5%	12.5%	14.4%
Prescription NSAIDs^b^		8.2%	8.1%	9.3%
OTC NSAIDs^b^		26.8%	26.7%	21.3%^a^
Coxibs^b^		10.2%	9.8%	4.7%^a^
Opioids^b^		3.3%	3.0%	5.0%
Chondroitin and/or Glucosamine^b^		40.7%	42.9%	35.4%^a^
Other nutraceutical^b^		8.6%	8.8%	10.2%
Intra-articular steroid injection^c^		4.2%	4.3%	7.3%
Intra-articular hyaluronan injection^c^		2.0%	1.7%	3.3%
Medications brought into visit or identifiedd:		N = 820	N = 690	N = 690
Proton pump inhibitor/H2 antagonist		11.2%	9.4%	21.4%
NSAID		7.7%	6.6%	13.9%
Coxib		11.6%	9.3%	5.2%
NSAID + PPI (% of NSAID users)		7.2% (6/83)	9.4% (5/53)	26.0% (25/96)
Coxib + PPI (% of coxib users)		34.9% (29/83)	30.7% (23/75)	41.7% (15/36)

^a^statistically significant change from baseline; ^b^for more than ½ days in the previous month; ^c^in the previous 6 months; din the previous month, brought in or identified in list of medication; Coxibs, COX-II inhibitors; KL, Kellgren-Lawrence; OTC, over the counter; NSAID, non-steroidal anti-inflammatory drug; NRS, numerical rating scale; PPI, proton pump inhibitor; SD, standard deviation.

Subjects were not specifically asked whether they were concurrently taking different medication types in the month prior to assessment; however, if they reported using two or more medication types for more than half of the days of the month (deemed frequent use), we inferred that these must have overlapped. The frequent use of a combination of conventional analgesic therapies in the month prior to enrolment was reported by 11.9% of participants at baseline (24.8% of those using an analgesic therapy). Combining analgesic therapies with nutraceuticals was also commonly reported (21.0%; see also Additional File 1, Table S1).

Of those who brought in or identified their specific medications in a list, use of a gastroprotective agent was reported by 11.2% (92/820) at baseline. Of the subjects listing a prescription NSAID (63/820; 7.7%), a proton pump inhibitor (PPI) or histamine (H2) receptor antagonist was listed by 7.2% (6/83), most commonly omeprazole or esomeprazole. Of those subjects who brought in or identified a coxib (95/820; 11.6%) amongst their prescription medications at baseline, 34.9% (29/83) also listed a PPI. A diagnosis of stomach ulcers or peptic ulcer disease was reported by 27 subjects. No subjects with stomach ulcer diagnosis reported use of prescription NSAIDs, five reported coxib use of whom four also used a PPI, whilst four reported OTC NSAID use with one also using a PPI.

### Factors associated with medication use at baseline

#### Any use of prescription analgesics

Use of prescription analgesics was more likely in female participants and in those with higher pain NRS scores but none of the other demographic or clinical variables were independently associated with prescription analgesic use (Tables 2 and 3). No interactions were found between pain and sex or race, or between age and presence of comorbidities or KL grade. Descriptive results for specific medication types (Table 4) suggest the observed trends were generally applicable to all prescription analgesics.

**Table 2:** Medication use during the 30 days prior to baseline, according to baseline demographic and clinical factors

			Medication type used within 30 days of baseline		
			
Variable			Analgesic(s)		Nutraceutical(s)
			
		n	Prescription; any use reported	Prescription or OTC; frequent use reported	Frequent use reported
Sex	Male	435	25.3%**	39.3%**	42.8%
	Female	552	33.9%**	54.5%**	40.2%
Race	White	701	28.7%	45.2%*	47.9%**
	Non-white	286	33.6%	54.2%*	25.2%**
Age	<65	594	29.6%	46.6%	40.9%
	65 to 74	312	29.5%	48.7%	44.2%
	>74	81	35.8%	53.1%	33.3%
KL grade	2	378	27.0%	43.9%	35.7%**
	3	442	31.7%	50.0%	41.0%**
	4	167	32.9%	50.9%	55.1%**
Pain NRS	0 to 3	233	22.7%**	32.6%**	47.6%*
	4 to 7	535	29.5%**	47.9%**	41.7%*
	8 to 10	219	39.3%**	63.9%**	33.8%*
Co-morb	Absent	696	28.7%	45.8%	45.7%**
	Present	291	33.3%	52.6%	30.9%**
BMI	<26	129	27.1%*	44.2%**	48.8%**
	26 to 30	381	26.2%*	42.3%**	45.4%**
	>30	477	34.0%*	53.2%**	36.1%**

BMI, body mass index kg/m^2^; Co-morb, co-morbidity; KL, Kellgren-Lawrence; NRS, numerical rating scale; OTC, over the counter. Unadjusted comparison significant at *P <0.05 or **P <0.01.

**Table 3:** Baseline demographic and clinical factors associated with medication use at baseline; results of multivariable modelling

	Outcome			
	
	Analgesic(s)			Nutraceutical(s)
		
Baseline factor	Prescription; any use reported	Prescription or OTC; frequent use reported	Prescription or OTC; number of different types used	Frequent use reported
Female	**1.5 (1.1 - 2.0), *P *= 0.010**	**1.8 (1.3 - 2.3), *P *>0.001**	**IR 1.4 (1.2 - 1.8), *P *= 0.001**	1.2 (0.9- 1.6), *P *= 0.197
Non-white	1.0 (0.7 - 1.4), p = 0.891	1.0 (0.7 - 1.3), p = 0.908	IR 1.0 (0.8 - 1.2), *P *= 0.733	**0.4 (0.3 - 0.6), *P *<0.001**
Age	reference	reference	reference	reference
65 to 74 versus <65	0.9 (0.7- 1.3), *P *= 0.722	1.0 (0.8 - 1.4), *P *= 0.919	IR 1.0 (0.8 - 1.2), *P *= 0.669	1.0 (0.8 - 1.4), *P *= 0.922
>74 versus <65	1.4 (0.8 - 2.3), *P *= 0.224	1.4 (0.8 - 2.3), *P *= 0.197	IR 1.0 (0.7 - 1.5), *P *= 0.830	**0.6 (0.3 - 0.9), *P *= 0.026**
KL grade	reference	reference	reference	reference
3 versus 2	1.2 (0.9 - 1.7), *P *= 0.180	1.2 (0.9 - 1.7), *P *= 0.133	IR 1.1 (0.9 - 1.4), *P *= 0.375	1.3 (0.9 - 1.7), *P *= 0.108
4 versus 2	1.4 (0.9 - 2.1), *P *= 0.107	1.4 (1.0 - 2.1), *P *= 0.071	IR 1.3 (0.9 - 1.7), *P *= 0.147	**2.2 (1.5 - 3.3), *P *<0.001**
Pain NRS	reference	reference	reference	reference
4 to 7 versus 0 to 3	1.3 (0.9 - 1.9), *P *= 0.152	**1.7 (1.2 - 2.4), *P *= 0.001**	IR 1.3 (1.0 - 1.7), *P *= 0.070	0.9 (0.6 - 1.2), *P *= 0.496
8 to 10 versus 0 to 3	**1.9 (1.2 - 2.9), *P *= 0.004**	**3.1 (2.1 - 4.7), *P *<0.001**	**IR 1.7 (1.3 - 2.4), *P *= 0.001**	0.8 (0.5 - 1.2), *P *= 0.204
Co-morbidity	1.1 (0.8 - 1.5), *P *= 0.544	1.1 (0.8 - 1.5), *P *= 0.491	IR 1.1 (0.9 - 1.4), *P *= 0.337	**0.6 (0.5 - 0.9), *P *= 0.005**
BMI	reference	reference	reference	reference
26 to 30 versus <26	1.0 (0.6 - 1.6), *P *= 0.944	1.0 (0.7 - 1.5), *P *= 0.973	IR 1.0 (0.7 - 1.4), *P *= 0.868	0.9 (0.6 - 1.4), *P *= 0.686
>30 versus <26	1.3 (0.9 - 2.1), *P *= 0.205	1.4 (0.9 - 2.1), *P *= 0.152	IR 1.2 (0.9 - 1.7), *P *= 0.191	0.7 (0.5 - 1.1), *P *= 0.114

Number of subjects included	987	987	987	987
Overall model significance: Likelihood ratio test	**χ^2^=30.3, df = 11, p = 0.001**	**χ^2^=72.7, df = 11, p<0.001**	**χ^2^=38.8, df = 11, p<0.001**	**χ^2^=81.2, df = 11, p<0.001**
Goodness-of-fit test: Hosmer & Lemeshow	χ^2^=2.3, df = 8, p = 0.970	χ^2^=11.27, df = 8, p = 0.187	χ^2^=564.6*, df = 975, p = 0.579	χ^2^=2.6, df = 8, p = 0.955

*Pearson Chi-square goodness-of-fit; Values presented are odds ratio (95% CI) unless otherwise stated; bold type indicates statistical significance. BMI, body mass index kg/m^2^; IR, incident rate; KL, Kellgren-Lawrence; NRS, numerical rating scale; OTC, over the counter.

**Table 4:** The proportions of participants using specific types of analgesics or nutraceuticals at baseline according to demographic and clinical characteristics

Variable		n	A'phen	Px NSAID	OTC NSAID	COXIB	Opoids	Gluco/chond	Other nutra
Sex	Male	435	8.3%	6.9%	25.7%	6.4%	1.4%	42.3%	9.0%
	Female	552	17.6%	9.2%	27.7%	13.2%	4.9%	39.5%	8.3%
Race	White	701	11.6%	8.0%	24.7%	11.7%	2.7%	47.4%	10.1%
	Non-white	286	18.2%	8.7%	32.2%	6.6%	4.9%	24.5%	4.9%
Age	<65	594	12.3%	10.1%	27.3%	9.3%	3.4%	40.1%	8.9%
	65 to 74	312	14.1%	6.1%	26.3%	11.9%	3.2%	43.9%	8.3%
	>74	81	19.8%	2.5%	25.9%	11.1%	3.7%	33.3%	7.4%
KL grade	2	378	12.4%	9.8%	22.2%	9.3%	3.7%	35.4%	7.1%
	3	442	13.6%	7.5%	30.5%	9.5%	3.6%	39.8%	7.9%
	4	167	15.6%	6.6%	27.5%	14.4%	1.8%	55.1%	13.8%
Pain NRS	0 to 3	233	6.9%	6.9%	19.7%	9.4%	0.4%	47.2%	7.7%
	4 to 7	535	14.8%	6.9%	26.5%	9.7%	3.0%	41.7%	8.4%
	8 to 10	219	17.4%	12.8%	35.2%	12.3%	7.3%	31.5%	10.0%
Co-morb	Absent	696	10.1%	7.6%	27.2%	10.6%	2.7%	44.8%	9.9%
	Present	291	21.6%	9.6%	26.1%	9.3%	4.8%	30.9%	5.5%
BMI	<26	129	11.6%	9.3%	24.0%	8.5%	2.3%	48.8%	10.0%
	26 to 30	381	9.7%	5.0%	24.9%	10.0%	1.6%	44.9%	9.7%
	>30	477	17.0%	10.5%	29.1%	10.9%	5.0%	35.2%	7.3%

A'phen, acetaminophen; BMI, body mass index kg/m^2^; chond, chondroitin; Gluco, glucosamine; KL, Kellgren-Lawrence; NRS, numerical rating scale; NSAID, non-steroidal anti-inflammatory drug; nutra, nutraceutical; OTC, over the counter; Px, prescription.

#### Frequent use of prescription or OTC analgesics

For frequent use of OTC or prescription analgesics the trends were generally consistent with those found for prescription analgesics (Tables 2 and 3). Frequent analgesic use was more likely in women and in subjects with higher pain NRS. No substantive interactions were found in this model.

Inspection of the descriptive data for specific medication types (Table 4) suggests that the lack of association between analgesic use and age when all types were combined together was not consistently observed for individual analgesic types; while use of prescription NSAIDs appeared to be less common in older patients, use of OTC NSAIDs was relatively consistent across the age groups, and acetaminophen use was more common in older patients.

Women and participants with higher pain NRS scores tended to use more types of prescription or OTC analgesics; no associations with race, age, KL grade, comorbidity or BMI were found and there were no substantive interactions (Table 3).

#### Frequent use of nutraceuticals

Frequent use of nutraceuticals was less likely in non-white participants and in those with comorbidities, and was more likely in those with KL grade 4 compared to KL grade 2 (Table 3). Those older than 74 years were significantly less likely to take nutraceuticals than those under 65. There was no evidence that gender, pain NRS or BMI were substantively associated with nutraceutical use. These trends were similar in participants taking glucosamine and/or chondroitin and those taking the less commonly used nutraceuticals (Table 4).

### Change in medication use over time

#### Changes at the cohort level

Compared to baseline, at 36 months there was a reduction in the number of participants using prescription analgesics in the previous month (29.4% versus 24.2%; McNemar's *P *= 0.004) and reporting frequent use of nutraceuticals (43.4% versus 36.0%; *P *<0.001), and fewer nutraceuticals were being used (standardised statistic = -3.71, *P *<0.001). Despite this, following correction for multiple comparisons there was no statistically significant reduction in the number reporting frequent use of any analgesic(s) (*P *= 0.013) and the total number of analgesic medication types used had not decreased significantly (Wilcoxon signed rank standardised statistic = -1.69, *P *= 0.091) (Table 1). The frequency of acetaminophen and prescription NSAID use remained at similar levels (acetaminophen, 12.5% versus 14.4%; prescription NSAIDs, 8.1% versus 9.3%). Reported use of OTC NSAIDs (26.7% versus 21.3%; McNemar's *P *= 0.004), coxibs (9.8% versus 4.7%; *P *<0.001) and chondroitin and/or glucosamine (42.9% versus 35.4%; *P *<0.001) was reduced. There was a substantial increase in the use of gastroprotective agents (9.4% versus 21.4%).

#### Changes at the level of the individual

Analysis of medication usage at an individual participant level indicated that the majority of people who were using medication at baseline had changed their medication type during the three year observation period (Table 5), although all of the participants who reported using an analgesic at baseline also reported using at least one analgesic at 36 months. Of those using a traditional analgesic at baseline, approximately one-third reported use of the same analgesic type at 36 months (ranging from 26.2% of baseline prescription NSAID users to 40.6% of baseline acetaminophen users). Nutraceuticals were more commonly continued, with 61.8% of participants using chondroitin and/or glucosamine at baseline still reporting use at 36 months. Of the 149 patients who reported using chondroitin at each of the annual follow-ups between baseline and 36 months, 99.3% also reported using glucosamine consistently over the same period. Changes in analgesic and nutraceutical use over 36 months according to demographic and clinical variables are presented in Additional File 1, Tables S2 and S3.

**Table 5:** Change in medication use by individuals between baseline and 36 months

	Proportion of baseline users (n/N)	
	
Therapy type used in month prior to visit	Used at both BL and 36 months	Used at BL but not 36 months
Acetaminophen	40.6% (41/101)	59.4% (60/101)
Prescription NSAIDs	26.2% (17/65)	73.8% (48/65)
OTC NSAIDs	39.1% (84/215)	60.9% (131/215)
Coxibs	31.6% (25/79)	68.4% (54/79)
Opioids	29.2% (7/24)	70.8% (17/24)
Chondroitin and/or glucosamine	61.8% (214/346)	38.2% (132/346)

BL, baseline; Coxibs, COX-II inhibitors; NSAID, non-steroidal anti-inflammatory drug; OTC, over the counter.

#### Subjects who consistently used no pain medication throughout follow-up

A total of 88 participants (10.9%) reported that they had not used any form of medication for pain, aching or stiffness for more than half the days of at least one month in the preceding year at each annual follow-up visit up until 36 months. We investigated the predictors of ever using any form of pain medication (not limited to prescription or OTC analgesics) during the follow-up period. Female subjects (OR 95% CI 1.2 to 3.2, *P *= 0.009) and those with moderate (1.6 to 2.6, *P *= 0.090) or severe (1.8 to 12.0, *P *= 0.002) baseline pain were more likely to use some form of pain medication (Table 6 and Additional File 1, Tables S2 and S3). Patients whose pain remained the same or worsened were more likely to take medication at some point during follow-up than those whose symptoms improved beyond smallest detectable difference (SDD) (1.0 to 2.8, *P *= 0.043). The odds of using pain medication were unchanged in participants 65- to 74-years old (0.5 to 1.3, *P *= 0.320) but were reduced to a borderline-significant degree in those more than 74 years old compared to those under 65 (0.2 to 1.0, *P *= 0.053). Patients with BMI >30 were more likely to use some form of medication than those with BMI <26 (1.2 to 4.7, *P *= 0.010). There was some indication that participants with KL grade 4 (1.0 to 4.3, *P *= 0.061) and those with comorbidities (1.0 to 3.2, *P *= 0.066) were more likely to use medication.

**Table 6:** Results of a multiple binary logistic regression model for use of medication for knee pain.

Variable		Ever used pain meds
Female		**1.9 (1.2 - 3.2), *P *= 0.009**
Non-white		1.2 (0.6 - 2.2), *P *= 0.671
Age		*P *= 0.136
	65 to 74 versus <65	0.8 (0.5 - 1.3), *P *= 0.320
	>74 versus <65	0.5 (0.2 - 1.0), *P *= 0.053
KL grade		*P *= 0.151
	3 versus 2	1.3 (0.8 - 2.2), *P *= 0.262
	4 versus 2	2.0 (1.0 - 4.3), *P *= 0.061
Pain NRS		***P *= 0.007**
	4 to 7 versus 0 to 3	1.6 (0.9 - 2.6), *P *= 0.090
	8 to 10 versus 0 to 3	**4.6 (1.8 - 12.0), *P *= 0.002**
Co-morbidity		1.8 (1.0 - 3.2), *P *= 0.066
BMI		***P *= 0.021**
	26 to 30 versus <26	1.3 (0.7 - 2.5), *P *= 0.360
	>30 versus <26	**2.4 (1.2 - 4.8), *P *= 0.010**
Pain NRS same^a ^or worse		**1.7 (1.0 - 2.8), *P*=0.043**

Number of patients included		806
Overall model significance: Likelihood ratio test		**χ^2^=53.4, df = 12, *P *<0.001**
Goodness-of-fit test: Hosmer & Lemeshow		χ^2^=5.48, df = 8, *P *= 0.713

^a^or improved by less than 2 units; BMI, body mass index kg/m^2^; KL, Kellgren-Lawrence; NRS, numerical rating scale; OTC, over the counter. For categorical variables the joint significance of the combined categories is presented in addition to the results for each category relative to the reference category. Values presented are odds ratio (95% CI) of reporting use of any form of medication for pain, aching or stiffness in either knee at any time during the 36-month follow-up period unless otherwise stated; values in bold type indicate statistical significance.

## Discussion

In this analysis of a large cohort with symptomatic radiographic knee OA, most people were using pharmacological therapies frequently. The most commonly used traditional analgesics were OTC NSAIDs, acetaminophen and prescription NSAIDs; within the limitations of this analysis, traditional analgesic use appeared to be in line with the American College of Rheumatology (ACR) guidelines which recommend the use of acetaminophen, NSAIDs and tramadol as first-line therapies for knee OA [15]. However the most commonly used overall therapy was nutraceuticals, despite a lack of recommendations for their use. Analysis of the medication types used by individuals for their joint pain revealed a high rate of change over three years, in line with previous reports that patients frequently change or discontinue treatments used for OA pain [33-35]. The relatively small proportion of people using an NSAID or an opioid at baseline who also reported use at 36 months may reflect a number of issues, including inadequate pain relief, side effects and clinician analgesic strategies (for example, switching agents to prevent development of tolerance to an agent). Notably, approximately two-thirds of participants using a nutraceutical at baseline were still using it at 36 months. Use of conventional analgesic combinations was uncommon, although use of an analgesic with a nutraceutical was reported by 21.0% of participants. Higher pain levels were associated with increased use of prescription and OTC analgesics.

Opioids were used infrequently. This is in contrast to a recent US study of medication usage in OA patients prior to total hip or total knee replacement, which reported opioids as the most commonly used medication [36]. Due to concerns about dependence and side effects, particularly in older individuals, opioids are often reserved for individuals with pain refractory to treatment with other medications and/or non-pharmacologic interventions. Hence, the low use of opioids observed in this study may reflect the relatively low pain scores in this OA population and the less advanced nature of the disease compared to the pre-joint replacement population. Although opioid use was slightly higher in the group excluded from the study due to knee replacement, it was still lower than that reported in previous studies [36]. The infrequent use of opioids may also reflect uncertainty on the role of opioids in musculoskeletal disorders and the lack of adequate training in these agents, as highlighted by a recent ACR Taskforce [37].

At 36 months there was a notable reduction in coxib usage which may reflect the withdrawal of rofecoxib and valdecoxib from the U.S. market. The decrease in coxib use at 36 months is likely reflective of both changes in medication availability and increasing awareness of toxicity during this period [24,26,38].

Use of gastroprotective agents with concomitant NSAID therapy was low at baseline (7.2%) and a higher incidence of gastroprotective agent use was seen in coxib users (34.9%). Almost one-fifth of subjects with a history of stomach ulcers reported coxib use (with three-quarters of these also using a PPI) compared to no reported use of prescription NSAIDs by subjects with a history of stomach ulcers or peptic ulcer disease. The high co-prescription of gastroprotective agents with coxibs may reflect prescription of coxibs to patients at higher risk of GI toxicity. At 36 months, use of gastroprotection was increased and, although still low, the proportion of NSAID users reporting use of gastroprotection was 3.5 times that at baseline. This could possibly reflect the awareness of findings supporting the cost-effectiveness of the addition of gastroprotective agents to both NSAIDs and coxibs [39].

Although prescription or OTC analgesic use was no more or less common in patients aged >74 when all drug types were considered simultaneously, there were apparently conflicting trends for specific analgesic types. There was a reduction in prescription NSAID use, which may reflect recent recommendations that oral NSAIDs should not be prescribed for those older than 75 years or with GI/CV risk [40]. This is in contrast to previous studies suggesting a high rate of NSAID prescription in populations with high GI and/or CV risk [27]. However there was an increase in acetaminophen use and, perhaps more worryingly, no reduction in OTC NSAID use in this population, suggesting that although practitioners may be adhering to guidelines recommending that NSAIDs are not prescribed to their older patients, these patients are still obtaining over-the-counter NSAIDs. It is of note that this group were more likely to report using no pain medication at all during follow-up (18.0% versus 9.7% of those less than 65 years old), which may reflect the tolerability and toxicity of current analgesic therapies.

Women were more likely to use prescription or OTC analgesics and used a greater number of different types, in line with previous reports which have found increased general prescription medication use in women, particularly those over the age of 65, compared to men. These studies also found women more likely to take multiple prescription medications in combination and more likely to take analgesic medications [22]. Increased odds of medication use may reflect reports of higher pain scores in women compared to men [10] although in our analyses female sex remained predictive of analgesic use even after controlling for pain.

Previous studies have reported higher OA-related pain scores in non-white study participants compared to white participants. Having controlled for pain, there were no differences observed in the reported use of analgesics; however, non-white participants were significantly less likely to use nutraceuticals than white participants. Decreased use of expensive OTC products may reflect differences in socio-economic status, with three times as many non-white subjects in the cohort reporting a total income <$25,000/year compared to white subjects (32.3% versus 11.1%).

This study has a number of limitations. Some patients had to be excluded from the analysis because their centralised KL reading was missing and we could not therefore confirm a diagnosis of radiographic OA. There was a high rate of loss to follow-up and incomplete data at 36 months, although the majority of characteristics were not associated with the likelihood of missing data or loss to follow-up. With the exception of PPI use, all medication use was self-reported. Although there was very good correlation between the reported use of coxibs and the identification of coxib medications [see Additional File 1, Methods], the correlation was much lower for prescription NSAID use; more subjects reported use than brought in or identified an NSAID. There were only a small number of people more than 74 years old; therefore, caution must be used in interpreting patterns of drug use in this group.

## Conclusions

In summary, we found that people with knee OA used pain medications frequently and generally in line with current guidelines from the ACR. Use of opioid medications was surprisingly low whilst the high frequency of medication change may indicate issues of lack of durability of efficacy and lack of long-term tolerability associated with conventional OA analgesics. The persistent use of OTC NSAIDs in the older population is an area of concern, given the enhanced risk of side effects and increased incidence of comorbidities.

## Abbreviations

ACR: American College of Rheumatology; BMI: body mass index; coxib: COX-II inhibitor; CV: cardiovascular; GI: gastrointestinal; KL: Kellgren-Lawrence; MSM: methylsulfonylmethane; NRS: numerical rating scale; NSAID: non-steroidal anti-inflammatory drug; OA: osteoarthritis; OAI: Osteoarthritis Initiative; OTC: over the counter; PPI: proton pump inhibitor; SAMe: S-adenosylmethionine; SDD: smallest detectable difference; TKR: total knee replacement.

## Competing interests

MH is a consultant to Astra Zeneca, Bioiberica, Covidien, Eli Lilly, Merck, NiCox, Pfizer, Pozen and Theralogix LLC. PGC receives research funding from Centocor and Pfizer and is a speaker or on advisory boards for AstraZeneca, Bioiberica, BMS, Merck, Novartis, Pfizer, Roche. SRK, EMAH and CAEW declare that they have no competing interests.

## Authors' contributions

PGC, SRK and CAEW conceived the study. PGC, MCH, SRK, EMAH and CAEW analysed and interpreted the data. SRK, EMAH and PGC drafted the manuscript. All authors read and approved the final manuscript.

## Supplementary Material

Additional file 1 **A supplementary file containing**. 1) Supplementary methods containing details of the OAI database, ethical approval and eligibility assessment, and OAI exclusion criteria. 2) Supplementary results containing details of the robustness of patient reported medication use and the combinations of medications used by participants. 3) Supplementary Table S1 showing combinations of analgesics and nutraceuticals taken by subjects at baseline. 4) Supplementary Table S2 showing changes in nutraceutical use at 36 months relative to baseline according to demographic and clinical variables and change in pain. 5) Supplementary Table S3 showing changes in analgesic medication use over 36 months according to demographic and clinical variables and change in pain. Click here for file
