# Supplementary material for: How do people with knee osteoarthritis use osteoarthritis pain medications and does this change over time? Data from the Osteoarthritis Initiative
Source: Arthritis Res Ther. 2013 Sep 4;15(5):R106. doi: 10.1186/ar4286 (PMC3978852; doi:10.1186/ar4286)
Supplement: Additional file 1 — A supplementary file containing. 1) Supplementary methods containing details of the OAI database, ethical approval and eligibility assessment, and OAI exclusion criteria. 2) Supplementary results containing details of the robustness of patient reported medication use and the combinations of medications used by participants. 3) Supplementary Table S1 showing combinations of analgesics and nutraceuticals taken by subjects at baseline. 4) Supplementary Table S2 showing changes in nutraceutical use at 36 months relative to baseline according to demographic and clinical variables and change in pain. 5) Supplementary Table S3 showing changes in analgesic medication use over 36 months according to demographic and clinical variables and change in pain. [file ar4286-S1.DOCX]

**Additional File 1**

**Methods**

The OAI database

Data used in this article were obtained from the AllClinical00, V0.2.2 and AllClinical05, V5.2.1 databases. Demographic data were obtained from the database Enrollees, V13. Kellgren-Lawrence grades were obtained from the database kXR_SQ_BU, which contains semi-quantitative readings of fixed-flexion knee X-rays from the Boston University X-ray reading centre. Prescription inventory data and gastroprotective medication use was taken from the MIF00 and MIF05 database.

Ethical approval and eligibility assessment

Ethical approval for the study was granted by the Committee on Human Research, University of California, San Francisco (IRB approval number 10-00532). Informed written consent was obtained from all subjects prior to inclusion. All patients consenting to participate in the OAI completed a standard group of self-report questionnaires during the eligibility, screening and enrolment (baseline) visits. The maximum time period between the initial telephone eligibility interview and screening and between the screening clinic visit and the enrolment (baseline) clinic visit was 6 weeks.

OAI exclusion criteria

Exclusion criteria include diagnosis of inflammatory arthritis, severity of joint space meaning participant was unlikely to demonstrate measurable loss of joint space during the study period (narrowing in both knees on baseline fixed flexion knee radiographs or unilateral joint replacement with severe joint space narrowing in other knee), bilateral knee joint replacement, unable to undergo 3.0Tesla MRI examination of the knee, positive pregnancy test, inability to provide blood sample, use of ambulatory aids other than a single straight cane for >50% of the time in ambulation, presence of co-morbidities that may interfere with likelihood of participation in 4-year long follow-up, unlikely to reside in the clinical area for at least 3 years, current participation in randomised control trial and unwillingness to sign informed consent.

**Results**

**Patterns of medication use at baseline**

Robustness of patient reported medication use

To confirm robustness of the medication data within the OAI database, we compared subject-reported NSAID and coxib use with the lists of medication (either brought in to visit or identified) for each participant. At baseline approximately half of subjects (820/987, 83.2% of the progression subcohort) brought in or identified all prescription medication taken in the 30 days preceding baseline. This increased to 85.6% (690/806) at 36 months. Use of a prescription coxib during the previous 30 days was reported by 113 subjects and 101 reported use more than half the days of the previous month, with 95 (84.1%) answering yes to both questions; 95 subjects brought in or identified a coxib amongst the medication used in the previous 30 days, all of whom had indicated use in the patient reported questionnaire. Use of a prescription NSAID during the previous 30 days was reported by 122 subjects and 81 reported use for more than half the days of the previous month, with 65 (53.3%) answering yes to both questions; 63 brought in or identified an NSAID amongst the medication used in the previous 30 days, all of whom had indicated use in the patient-reported questionnaire.

Combinations of medications

The most common combination was acetaminophen with OTC NSAIDs, taken by 5.0% of participants (Table S1). The combination of OTC and prescription NSAIDs was reported by 2.6%, whilst the use of coxibs with either OTC NSAIDs or acetaminophen was reported by 2.0% and 2.2% of participants, respectively. Use of an NSAID and/or coxib together with a nutraceutical was the most frequently reported combination therapy, taken by 17.9% of participants (85.5% of those using a combination of analgesic and nutraceuticals) compared to 5.4% taking a combination of acetaminophen with a nutraceutical (although almost two-thirds of these were also taking at least one other analgesic) and 1.2% combining an opioid with a nutraceutical. Of those taking nutraceuticals 87.7% reported use of both glucosamine and chondroitin, with 16.2% also using MSM.

**Table S1**: Combinations of analgesics and nutraceuticals taken by subjects at baseline

|  | **A’phen** | **OTC NSAID** | **Px NSAID** | **COXIB** | **Opoids** | **SAME** | **MSM** | **Doxy** | **Chond** | **Gluco** |
| --- | --- | --- | --- | --- | --- | --- | --- | --- | --- | --- |
| **A’phen** |  | 5.0%  (49) | 1.4%  (14) | 2.2%  (22) | 1.3%  (13) | 0.2%  (2) | 1.0%  (10) | 0.1%  (1) | 4.8%  (47) | 5.0%  (49) |
| **OTC NSAID** |  |  | 2.6%  (26) | 2.0%  (20) | 0.8%  (8) | 0.4%  (4) | 2.0%  (20) | 0.1%  (1) | 11.3%  (112) | 12.4%  (122) |
| **Px NSAID** |  |  |  | 0.4%  (4) | 0.4%  (4) | 0.2%  (2) | 0.8%  (8) | 0.2%  (2) | 2.7%  (27) | 3.3%  (33) |
| **COXIB** |  |  |  |  | 1.1%  (11) | 0.2%  (2) | 1.9%  (19) | 0.2%  (2) | 4.1%  (40) | 4.9%  (48) |
| **Opoids** |  |  |  |  |  | 0.1%  (1) | 0.4%  (4) | 0.2%  (2) | 0.8%  (8) | 0.9%  (9) |
| **SAME** |  |  |  |  |  |  | 0.4%  (4) | 0.1%  (1) | 0.7%  (7) | 0.8%  (8) |
| **MSM** |  |  |  |  |  |  |  | 0.2%  (2) | 6.7%  (66) | 7.4%  (73) |
| **Doxy** |  |  |  |  |  |  |  |  | 0.1%  (1) | 0.2%  (2) |
| **Chond** |  |  |  |  |  |  |  |  |  | 36.3%  (358) |

**Table S2:** Changes in nutraceutical use at 36 months relative to baseline according to demographic and clinical variables and change in pain.

|  |  | **Frequent nutraceutical use in the month prior to visit** | | | | | |
| --- | --- | --- | --- | --- | --- | --- | --- |
|  | **n** | **None used** | **Used at 36m but not BL** | **Same types used** | **Switched types** | **More types at 36m** | **Fewer types at 36m** |
| **Sex Male** | 361 | 39.6% | 9.7% | 9.1% | 11.6% | 7.8% | 22.2% |
| **Female** | 445 | 43.6% | 8.1% | 8.3% | 13.5% | 6.1% | 20.4% |
| **Race White** | 591 | 35.9% | 9.3% | 11.3% | 13.0% | 7.3% | 23.2% |
| **Non-white** | 215 | 58.1% | 7.4% | 1.4% | 11.6% | 5.6% | 15.8% |
| **Age <65** | 486 | 41.8% | 8.2% | 7.2% | 14.4% | 6.2% | 22.2% |
| **65-74** | 259 | 39.8% | 9.7% | 11.6% | 10.8% | 8.1% | 20.1% |
| **>74** | 61 | 50.8% | 9.8% | 8.2% | 6.6% | 6.6% | 18.0% |
| **KL grade 2** | 306 | 44.8% | 8.8% | 7.8% | 14.4% | 4.6% | 19.6% |
| **3** | 366 | 44.0% | 8.5% | 9.0% | 12.3% | 6.6% | 19.7% |
| **4** | 134 | 29.1% | 9.7% | 9.7% | 9.7% | 12.7% | 29.1% |
| **Pain NRS 0-3** | 188 | 37.8% | 9.0% | 11.2% | 14.4% | 9.0% | 18.6% |
| **4-7** | 455 | 40.7% | 9.0% | 9.2% | 13.6% | 5.3% | 22.2% |
| **8-10** | 163 | 49.7% | 8.0% | 4.3% | 8.0% | 8.6% | 21.5% |
| **Co-morb Absent** | 580 | 36.7% | 10.2% | 9.7% | 12.8% | 8.3% | 22.4% |
| **Present** | 226 | 54.9% | 5.3% | 6.2% | 12.4% | 3.1% | 18.1% |
| **BMI <26** | 110 | 38.2% | 10.0% | 10.0% | 9.1% | 6.4% | 26.4% |
| **26-30** | 315 | 37.8% | 8.6% | 9.2% | 13.0% | 8.9% | 22.5% |
| **>30** | 381 | 46.2% | 8.7% | 7.9% | 13.4% | 5.2% | 18.6% |
| **Change in Better**^†^ | 266 | 44.0% | 8.6% | 8.6% | 10.9% | 6.0% | 21.8% |
| **pain NRS Same/worse** | 540 | 40.7% | 8.9% | 8.7% | 13.5% | 7.2% | 20.9% |
| **TOTAL** | 806 | 41.8% | 8.8% | 8.7% | 12.7% | 6.8% | 21.2% |

^†^by at least 2 units; BL, baseline; BMI, body mass index kg/m^2^; Co-morb, co-morbidity; KL, Kellgren-Lawrence; NRS, numerical rating scale

**Table S3:** Changes in analgesic medication use over 36 months according to demographic and clinical variables and change in pain.

|  |  | **Frequent prescription/OTC analgesic use in the month prior to visit** | | | | | | **Ever used any pain meds*** |
| --- | --- | --- | --- | --- | --- | --- | --- | --- |
|  | **n** | **None used** | **Used at 36m but not BL** | **Same types used** | **Switched types** | **More types at 36m** | **Fewer types at 36m** |  |
| **Sex Male** | 361 | 34.6% | 13.3% | 4.4% | 22.7% | 4.2% | 20.8% | 85.0% |
| **Female** | 445 | 25.4% | 12.6% | 5.2% | 24.0% | 7.2% | 25.6% | 92.4% |
| **Race White** | 591 | 32.5% | 13.0% | 5.4% | 22.3% | 4.7% | 22.0% | 87.5% |
| **Non-white** | 215 | 21.4% | 12.6% | 3.3% | 26.5% | 8.8% | 27.4% | 93.5% |
| **Age <65** | 486 | 29.8% | 13.6% | 4.9% | 22.0% | 6.4% | 23.3% | 90.3% |
| **65-74** | 259 | 30.1% | 12.7% | 5.4% | 25.1% | 4.2% | 22.4% | 88.4% |
| **>74** | 61 | 24.6% | 8.2% | 1.6% | 27.9% | 8.2% | 29.5% | 82.0% |
| **KL grade 2** | 306 | 34.6% | 12.1% | 3.9% | 22.9% | 4.2% | 22.2% | 86.6% |
| **3** | 366 | 27.0% | 13.1% | 5.7% | 21.9% | 7.9% | 24.3% | 90.2% |
| **4** | 134 | 24.6% | 14.2% | 4.5% | 29.1% | 3.7% | 23.9% | 91.8% |
| **Pain NRS 0-3** | 188 | 43.6% | 13.3% | 4.3% | 19.1% | 3.2% | 16.5% | 82.4% |
| **4-7** | 455 | 27.5% | 14.5% | 5.1% | 24.0% | 4.8% | 24.2% | 89.2% |
| **8-10** | 163 | 19.0% | 8.0% | 4.9% | 27.0% | 11.7% | 29.4% | 96.3% |
| **Co-morb Absent** | 580 | 32.2% | 12.2% | 5.3% | 21.7% | 5.5% | 22.9% | 87.6% |
| **Present** | 226 | 22.6% | 14.6% | 3.5% | 27.9% | 6.6% | 24.8% | 92.9% |
| **BMI <26** | 110 | 38.2% | 8.2% | 6.4% | 25.5% | 5.5% | 16.4% | 82.7% |
| **26-30** | 315 | 34.9% | 13.7% | 3.8% | 20.0% | 4.4% | 23.2% | 85.7% |
| **>30** | 381 | 22.6% | 13.6% | 5.2% | 25.7% | 7.1% | 25.7% | 93.7% |
| **Change in Better**^†^ | 266 | 31.6% | 9.4% | 4.5% | 24.4% | 4.1% | 25.9% | 86.8% |
| **pain NRS Same/worse** | 540 | 28.5% | 14.6% | 5.0% | 23.0% | 6.7% | 22.2% | 90.2% |
| **TOTAL** | 806 | 29.5% | 12.9% | 4.8% | 23.4% | 5.8% | 23.4% | 10.9% |

*Subject reported using any form of pain medication (not limited to prescription or OTC analgesics) for knee symptoms for at least half the days of one month during the previous year at each annual follow-up visit between baseline and 36 months; ^†^by at least 2 units; BL, baseline; BMI, body mass index kg/m^2^; Co-morb, co-morbidity; KL, Kellgren-Lawrence; NRS, numerical rating scale; OTC, over the counter
